# Congenital Vallecular Cyst: A Case Report of rare cause of upper air way obstruction in infant

**DOI:** 10.12669/pjms.40.6.9433

**Published:** 2024-07

**Authors:** Iram Iqbal, Ayaz Ur Rehman, Ariba Siddiqui, Naveed Ur Rehman Siddiqui

**Affiliations:** 1 Iram Iqbal, FCPS Department of Pediatric Medicine and Child Health, Aga Khan University Hospital, Karachi, Pakistan; 2 Ayaz Ur Rehman, MBBS Department of Pediatric Medicine and Child Health, Aga Khan University Hospital, Karachi, Pakistan; 3 Ariba Siddiqui, MBBS Department of Pediatric Medicine and Child Health, Aga Khan University Hospital, Karachi, Pakistan; 4 Naveed Ur Rehman Siddiqui, FCPS Department of Pediatric Medicine and Child Health, Aga Khan University Hospital, Karachi, Pakistan

**Keywords:** Vallecular cyst, Upper air way obstruction, Laryngeal cyst

## Abstract

Congenital vallecular cyst is one of the rare etiologies of upper airway obstruction. Due to the scarcity of literature review, the exact incidence is not known. We report the case of a 10-month-old infant, who came to to Aga Khan University Hospital (AKUH) for the first time with signs of upper airway obstruction; was initially misdiagnosed as foreign body aspiration for which an emergency bronchoscopy was performed that did not reveal any foreign body. The patient was then managed in the pediatric intensive care unit, where he was diagnosed as a congenital vallecular cyst on a subsequent laryngoscopy after extubation failure. The cyst was aspirated and cauterized by the ENT team. The patient was successfully extubated without any signs of upper airway obstruction. In evaluating a child with signs and symptoms of upper airway obstruction, it is crucial to consider not only common causes like foreign body, acute epiglottitis, and croup, but also rare factors such as laryngeal cysts.

## INTRODUCTION

Vallecular cyst is a rare condition and has been reported infrequently in the literature. However, it can have very important clinical implications. The small cyst may be asymptomatic, but when it increases in size due to infection or obstruction, it eventually fills the vallecula, causing dysphagia, odynophagia, life-threatening obstruction of the upper airways, and significant respiratory distress.^1^,^2^ The most common site is the lingual surface of the epiglottis. The prevalence and incidence of vallecular cyst is not exactly known; an estimated incidence of about 1.82 to 3.49 per 100,000 live births has been mentioned in literature.^2^ It is usually diagnosed incidentally during intubation for surgical procedures or for obstruction of the upper airways and, most of times causes difficult intubation.^3^ Fibreoptic laryngoscopy is the frequently used diagnostic technique for vallecular cysts. Complete surgical excision of the cyst is the treatment of choice.^4^

## CASE PRESENTATION

We present a case report of a 10-month-old male, vaccinated, developmentally normal, with no known comorbidities. He presented to the Emergency Department with a history of low-grade fever and cough from two days, breathing difficulty, and noisy breathing from one day. There was no history of flue, rash, choking, or foreign body inhalation. Previously, he had one hospital admission for three days for pneumonia at the age of three months. The parents were first cousins and there was no significant family history. On examination he was hemodynamically stable, but had stridor, signs of respiratory distress, and decreased intensity of breath sounds on the left side along with bilateral wheezing.

**Fig.1:**
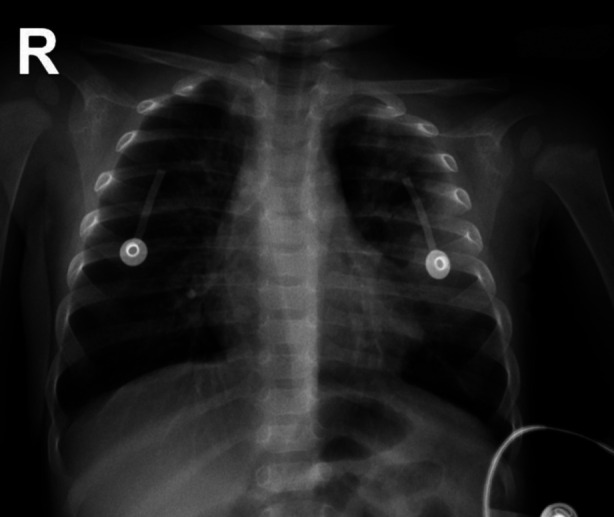
Frontal chest view X-ray showing non-homogeneous opacity in the left upper and middlezone.

The rest of the systemic examination was normal. The chest radiograph showed non-homogeneous opacity in the left upper and middle zones (Fig.1). The laboratories showed Hb of 8.9 g/dl, TLC counts of 13 x10 9 with neutrophils 86.1 %, lymphocytes 11, procalcitonin 2 ng / ml, and blood and tracheal aspirate cultures negative. His arterial blood gas showed mixed respiratory and metabolic acidosis. On the basis of this history, examination and radiographic findings, a provisional diagnosis of foreign body aspiration was made. The patient started to feel dehydrated and became drowsy and was intubated by the ER team.

**Fig.2:**
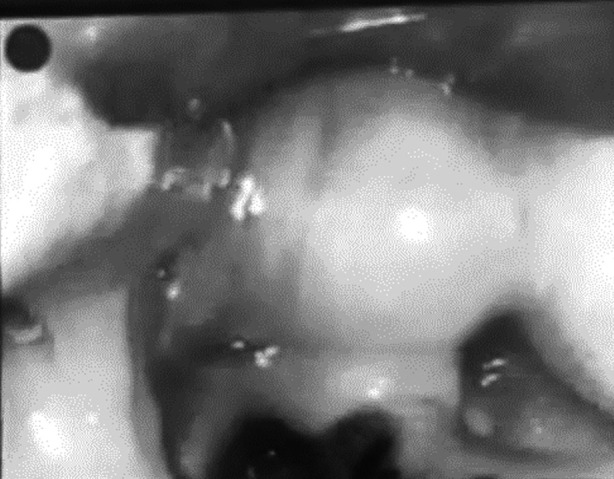
showing the vallecular cyst during reintubation with a C-MAC video laryngoscope.

Pediatric surgery and PICU teams were brought in and the patient was rushed to the operating room, where bronchoscopy was performed but no foreign body was visualized. The. Patient was transferred to the pediatric intensive care unit, where he initially required high ventilatory parameters, which were gradually weaned off. The patient tolerated spontaneous breathing tests and remained in pressure support mode of ventilation and was extubated on the second post-procedure day. The patient immediately developed stridor and respiratory distress and was reintubated by the PICU team using a MAC video laryngoscope, which showed a large cyst that obstructs the laryngeal inlet (Fig.2).

The vocal cords and the subglottic area appeared normal. The upper airway was reevaluated by the ENT consultant with a fibreoptic laryngoscope and a diagnosis of vallecular cyst was confirmed. Cyst was aspirated and cauterized by the ENT team in the operating room and the patient was shifted to PICU. Analgesics, antibiotics, and dexamethasone were administered postoperatively, and the next day was successfully extubated, without work of breathing or stridor. He did not require any noninvasive ventilation and was discharged home on the fifth postoperative day.

## DISCUSSION

Congenital cysts of the larynx are a rare cause of upper airway obstruction in infants and children. Laryngeal cysts are classified as ductal and saccular cysts. Ductal cysts result from obstruction and retention of mucus in the collecting ducts of the submucosal glands, whereas saccular cysts originate from the saccule and extend to the ventricle. The most common form of laryngeal cysts are the ductal cysts, comprising 75% of cases. Vallecular cysts account for 10 % 0f all laryngeal cysts.^5^ A vallecular cyst is a ductal cyst, which is a unilocular cystic mass arising most commonly from the lingual surface, the aryepiglottic fold, the ventricle, or the pyriform sinus as a result of ductal obstruction or embryologic malformation.^2^ Obstruction of the duct may be caused by inflammation, irritation, or trauma.

Vallecular cysts are generally located on the lingual surface of the epiglottis. Histologically, the cyst contains a respiratory epithelium with mucous glands, lined by squamous epithelium externally.^6^ Vallecular cyst may present at any age, a bimodal age of presentation has been mentioned, suggesting two clinical forms, the adult and pediatric vallecular cyst, each with a different pathogenesis. It is most prevalent in neonates and infants and has a sporadic occurrence. The vallecular cyst can become infected, resulting in acute epiglottitis or abscess formation, edema and inflammation of the surrounding laryngeal / pharyngeal structures, and subsequent life-threatening acute upper airway obstruction.^4^,^7^ However, the most common case of stridor in infants is laryngomalacia. Vallecular cyst often coexists with laryngomalacia. In 12-45% of patients with laryngomalacia, an associated airway abnormality like laryngeal cyst also exists.^8^ The vallecular cyst is mostly diagnosed with fibreoptic laryngoscopy. However, cervical ultrasound, CT and magnetic resonance imaging have also been used to rule out other differential diagnoses such as vallecular cyst, dermoid cyst, lingual thyroid, thyroglossal duct cyst and vascular anomalies and may be needed in the case of a small or occult cyst that cannot be easily recognized by fibreoptic endoscopy or in patients with recurrent epiglottitis.^9^

Various treatment modalities for a vallecular cyst include cyst aspiration, marsupialization, surgical excision, excision with CO2 laser or electrocautery, but complete surgical excision is the treatment of choice for a vallecular cyst, as all other techniques have a high recurrence rate. The prognosis is good with a low recurrence rate.

In our case, no imaging studies were performed as the diagnosis was obvious on laryngoscopy, so we proceeded with surgical management. ENT consultation could have been done earlier when the bronchoscopy did not reveal any foreign body in our case. We have also come to the conclusion that the use of the C-MAC video laryngoscope contributed to the diagnosis of vallecular cyst, which was initially missed on initial intubation with conventional laryngoscopy.

## CONCLUSION

We conclude that, in addition to considering foreign body and infectious causes of upper airway obstruction such as acute epiglottitis and croup, rare causes such as laryngeal cysts should also be taken into account when a child presents signs and symptoms of upper airway obstruction.
